# CD147 at the crossroads of glycoprotein networks, metabolic reprogramming, and metastatic progression

**DOI:** 10.3389/fimmu.2025.1727125

**Published:** 2025-12-17

**Authors:** Tz-Syuan Su, Jiunn-Wei Wang, Yu-Li Su, Ming-Hong Lin, Hsin-Ying Clair Chiou

**Affiliations:** 1 School of Medicine, College of Medicine, Kaohsiung Medical University, Kaohsiung, Taiwan; 2 Graduate Institute of Clinical Medicine, College of Medicine, Kaohsiung Medical University, Kaohsiung, Taiwan; 3 Department of Internal Medicine, Faculty of Medicine, College of Medicine, Kaohsiung Medical University, Kaohsiung, Taiwan; 4 Division of Gastroenterology, Department of Internal Medicine, Kaohsiung Medical University Hospital, Kaohsiung, Taiwan; 5 Division of Hematology Oncology, Department of Internal Medicine, Kaohsiung Chang Gung Memorial Hospital and Chang Gung University, College of Medicine, Kaohsiung, Taiwan; 6 School of Medicine, College of Medicine, National Sun Yat-sen University, Kaohsiung, Taiwan; 7 Institute for Translational Research in Biomedicine, Kaohsiung Chang Gung Memorial Hospital, Kaohsiung, Taiwan; 8 Genomic and Proteomic Core Laboratory, Department of Medical Research, Kaohsiung Chang Gung Memorial Hospital, Kaohsiung, Taiwan; 9 Department of Microbiology and Immunology, School of Medicine, College of Medicine, Kaohsiung Medical University, Kaohsiung, Taiwan; 10 Graduate Institute of Medicine, College of Medicine, Kaohsiung Medical University, Kaohsiung, Taiwan; 11 Department of Medical Research, Kaohsiung Medical University Hospital, Kaohsiung, Taiwan; 12 Department of Applied Chemistry, National Chi Nan University, Nantou, Taiwan

**Keywords:** cancer metabolism, CD147, CD44, pre-metastatic niche formation, tumor microenvironment

## Abstract

CD147 (also known as EMMPRIN or Basigin), a transmembrane glycoprotein of the immunoglobulin superfamily, functions as a pivotal regulator of tumor progression. It coordinates key oncogenic processes—including metabolic adaptation, chemoresistance, angiogenesis, and immune modulation—through an extensive network of protein–protein interactions. Metabolic reprogramming not only reshapes the intrinsic metabolic circuitry of tumor cells but also promotes the establishment of a pre-metastatic niche that facilitates metastatic seeding and outgrowth via dynamic metabolic crosstalk with immune and stromal components. Here, we review current evidence showing that CD147 mediates PMN formation by promoting immune evasion, metabolic adaptation, and stromal remodeling. Through the coordination with membrane-associated glycoproteins—including CD44, epidermal growth factor receptor (EGFR), integrins, CD280 (uPARAP/Endo180/MRC2), and CD276, CD147 orchestrates intracellular signaling events that drive cancer cell metabolic adaptation. These interactions contribute to metabolic reprogramming across glucose, lipid, amino acid, and mitochondrial pathways, thereby linking CD147-mediated metabolic plasticity to tumor dissemination and metastasis. By integrating insights into immune and stromal modulation within the tumor microenvironment (TME), this review highlights the multifaceted roles of CD147 and its glycoprotein interactome in shaping the metastatic niche.

## 1 Introduction

Cancer metastasis accounts for the majority of cancer-related deaths (1–3), with the average median survival for metastatic cancer patients being approximately 10 months (4). While the genetic and epigenetic evolution of cancer cells is essential for metastatic competence, increasing attention has turned to the non-malignant components of the tumor microenvironment (TME), particularly stromal and immune mediators that facilitate the formation of pre-metastatic niche (PMN) (5–7), which further provide a hospitable landscape for subsequent cancer cell colonization and outgrowth. The concept of PMN underscores the systemic nature of cancer progression, where soluble factors and extracellular vesicles (EVs) released by the primary tumors circulate in the bloodstream and prime distant tissues by attracting bone marrow-derived cells (BMDCs), reprogramming resident stromal and immune cells, increasing vascular permeability, and remodeling the extracellular matrix (ECM) (5–9). These changes collectively promote immune tolerance, angiogenesis, and metabolic adaptations in secondary organs—features that necessitate the survival of circulating tumor cells (CTCs).

Previous research has highlighted the roles of glycoproteins in orchestrating these systemic alterations. Glycoproteins are key mediators of cell adhesion, signal transduction, and immune modulation. Cancer-associated glycosylation changes significantly alter the behavior and function of these proteins, allowing tumor cells to escape immune surveillance, resist apoptosis, and interact with the ECM more effectively (10–12). Of particular interest is CD147 (also known as EMMPRIN or basigin), a transmembrane glycoprotein of the immunoglobulin superfamily (IgSF) that is overexpressed in many malignancies and contributes to metastasis through multiple mechanisms, including the activation of matrix metalloproteinases (MMPs), stimulation of vascular endothelial growth factor (VEGF) and hyaluronan production, facilitation of metabolic reprogramming, and promotion of leukocyte and platelet adhesion (13–18). Clinically, CD147 is implicated in a wide spectrum of tumors (19–24), where its expression is often correlated with tumor aggressiveness, metastatic potential, and poor prognosis.

The structural flexibility of CD147 enables dynamic interactions with various membrane-bound glycoproteins and receptors, such as CD44, epidermal growth factor receptor (EGFR), integrins, CD276, and CD280 (16, 25). These interactions activate diverse signaling cascades, including SAPK/JNK (26), PI3K/Akt/mTOR (27), and MAPK/ERK pathways (28), each contributing to distinct aspects of cancer cell survival, immune evasion, stromal activation, and metastatic colonization. For instance, CD147-CD44 interactions promote stem cell characteristics and epithelial-mesenchymal transition (EMT), while CD147-integrin complexes regulate platelet aggregation and leukocyte recruitment—both crucial for metastatic niche priming.

Despite growing interest in CD147’s oncogenic functions, the mechanistic underpinnings of its protein-protein interactions within immune and stromal contexts remain incompletely understood, particularly in the early events of pre-metastatic niche establishment. Given the role of these niches as gateways to metastasis, further elucidation of CD147’s involvement in shaping immune and stromal dynamics is both timely and necessary. This review aims to dissect the multifaceted functions of CD147 and its glycoprotein partners in modulating the tumor microenvironment during metastatic progression. By integrating molecular mechanisms with emerging clinical relevance, we highlight current challenges and the potential of targeting CD147-centered glycoprotein networks to disrupt niche formation, thus further improving clinical outcomes for advanced cancer patients.

## 2 Metabolic reprogramming in the tumor microenvironment and pre-metastatic niche

Cancer metabolism, first unveiled by Otto Warburg in the early 1920s through his observation that, despite adequate oxygen availability, cancer cells tend to favor aerobic glycolysis rather than oxidative phosphorylation (OXPHOS)—a metabolic reprogramming known as the Warburg effect (29). This metabolic reconfiguration sustains the energy production and anabolic processes required for uncontrolled tumor growth and is now recognized as a hallmark of cancer (30). The shift in metabolic pathways reflects not only intrinsic oncogenic alterations but also an adaptation to the hostile TME—characterized by hypoxia, acidosis, oxidative stress, and nutrient deprivation—where metabolic plasticity enables cancer cells to survive, proliferate, and metastasize under otherwise inhospitable conditions (31–35). This concept resonates with the “seed and soil” hypothesis proposed by Stephen Paget in 1889, which posits that the success of metastasis depends on the compatibility between disseminated tumor cells (the “seed”) and the organ-specific microenvironment (the “soil”) (36, 37). In glucose metabolism, the enhanced expression of glucose transporters such as GLUT1 and glycolytic enzymes including hexokinase 2 (HK2), phosphofructokinase (PFK), and pyruvate kinase M2 (PKM2) allows cancer cells to increase glucose uptake and flux through glycolysis, generating lactate as a major end product. Although less efficient in ATP production, this metabolic pathway facilitates the swift generation of intermediates essential for the synthesis of nucleotides, amino acids, and lipids. Alterations in the tricarboxylic acid (TCA) cycle, including suppression of succinate dehydrogenase (SDH) and accumulation of intermediates such as succinate, fumarate, and citrate, further support tumor proliferation by contributing to anabolic metabolism and epigenetic regulation (30, 38–40). In parallel, fatty acid metabolism is reprogrammed to favor *de novo* lipogenesis, supplying essential components for energy storage, membrane biosynthesis, and signal transduction. Upregulation of ATP-citrate lyase (ACLY), acetyl-CoA carboxylase (ACC), and fatty acid synthase (FASN) is commonly observed in tumors, facilitating the conversion of citrate to acetyl-CoA, then to malonyl-CoA, and ultimately to palmitate. This enhanced lipogenesis supports tumor progression and contributes to therapeutic resistance (38, 41, 42). Amino acid metabolism is also essential, with glutamine acting as a pivotal nutrient that fuels the TCA cycle, supports nucleotide and lipid synthesis, and maintains redox homeostasis. Through glutaminolysis, glutamine contributes to the activation of the mammalian target of rapamycin complex 1 (mTORC1), promoting cancer cell proliferation and inhibiting apoptosis (30, 43). Proline, a non-essential amino acid, plays a crucial role in redox balance and adaptive stress responses, enabling tumor cells to withstand unfavorable conditions (30); additionally, its abundance in collagen—a major component of the ECM—implies that ECM degradation not only releases proline as an energy substrate but also promotes tissue remodeling and metastasis (44). Under hypoxic conditions, the availability of aspartate and asparagine becomes a limiting factor for cell proliferation; however, cancer-associated fibroblasts (CAFs) can compensate by supplying these amino acids to tumor cells via transporters such as SLC1A3, thereby promoting protein and nucleotide synthesis (44). This metabolic symbiosis between stroma and tumor cells exemplifies the reciprocal nature of metabolic remodeling within the TME. CAFs not only secrete amino acids but also reshape the ECM and secrete pro-tumorigenic cytokines, while metabolites like glutamine and arginine from cancer cells support proline synthesis and ECM deposition in CAFs (44). Collectively, these metabolic alterations not only sustain the bioenergetic and anabolic needs of tumor cells but actively reprogram the TME, enhancing angiogenesis, immunosuppression, fibrosis, and ultimately metastasis (45).

## 3 CD147 as a molecular mediator of pre-metastatic niche formation

CD147 is encoded by the BSG gene on chromosome 19p13.3 and comprises 269 amino acids, including a signal peptide, an extracellular domain, a transmembrane domain, and a short cytoplasmic tail. The protein contains three potential N-linked glycosylation sites on asparagine residues, which significantly influence its structure and function (23, 46–48). There are four isoforms of CD147 in humans. Basigin-1, a retina-specific receptor for rod-derived cone viability factor (RdCVF), consists of three immunoglobulin-like (Ig-like) domains: D0, D1, and D2. In contrast, basigin-2, the predominant isoform ubiquitously expressed across tissues and primarily involved in tumor progression, contains two Ig-like domains (D1 and D2) separated by a flexible five-amino-acid linker featuring a conserved Gly-Pro-Pro (GPP) motif (16, 25). Crystallographic studies of basigin-2 have revealed a distinctive arrangement of its extracellular domains, diverging from the classical V-set domain configuration typical of IgSF members. Instead, basigin-2 adopts a C2-I domain configuration, enabling both homophilic (C2-C2) and heterophilic (C2-I) dimerization. These interfaces are crucial for CD147’s ability to mediate protein–protein interactions, facilitating the formation of homo-oligomers and higher-order complexes. This atypical structural organization underpins its diverse functional roles, particularly in regulating tumor cell behavior, inflammatory signaling, and extracellular matrix remodeling (25). Basigin-3 and basigin-4, broadly expressed in normal tissues and upregulated in HCC, are transcribed from an alternative promoter. Although both isoforms localize to the plasma membrane, only basigin-3 is detectable at the protein level in cultured cells. Notably, basigin-3 forms hetero-oligomers with basigin-2, thereby inhibiting HCC cell progression and MMP induction—functioning as an endogenous inhibitor of basigin-2’s pro-tumorigenic activity (49). CD147 exists not only in its membrane-anchored form but also as a soluble form released into the extracellular environment (47, 50). Soluble CD147 has been identified in various biological fluids, including ocular and synovial fluids, plasma, and platelet releasates, as well as in the conditioned media of tumor cells such as HEp-2 human laryngeal epidermoid carcinoma cells (47). This soluble form is mainly generated through proteolytic shedding of the extracellular domain of membrane-bound CD147 or through the release of CD147-containing microvesicles and subsequent cleavage (50). Functionally, soluble CD147 acts in a paracrine manner by binding to membranous CD147 on neighboring cells, thereby promoting its dimerization and activating downstream signaling cascades that enhance tumor cell proliferation, migration, and angiogenesis (50). In HEp-2 cells, soluble CD147 stimulates MMP production in fibroblasts, consequently facilitating cancer cell invasion and metastatic dissemination (51). In contrast, in HNSCC cell lines, soluble CD147 mediates neutrophil activation, suggesting its broader role in modulating the TME (52).

This structural feature confers conformational flexibility to the extracellular region of CD147, enabling dynamic interdomain interactions and positioning CD147 as a multifunctional signaling platform through its engagement with numerous binding partners. These include EGFR, GLUT1, MCTs (MCT1 and MCT4), integrins, CD44, and the calcium-binding protein S100A9. Collectively, these interactions regulate key cancer-related processes such as metabolic reprogramming, matrix degradation, proliferation, immune modulation, angiogenesis, and cell migration (16). Aberrant expression and activation of CD147 have been documented in a wide range of malignancies, including HNSCC (53, 54), NSCLC (19), BC (20), EC (21, 55), HCC (22, 23), and BLCA (24). In HNSCC, CD147 promotes tumor progression through activation of the nuclear factor-κB (NF-κB) signaling pathway (53) and enhances resistance to chemotherapeutic agents via the MAPK/ERK cascade (54). In BC, CD147 facilitates EMT, thereby enhancing migratory and invasive capabilities through MAPK/ERK signaling (28). In NSCLC, CD147 forms a complex with CD98 heavy chain, activating the phosphoinositide 3-kinase (PI3K)/Akt pathway and promoting cellular proliferation (56). In HCC, CD147 orchestrates a network of pro-metastatic signals, including the TGF-β, MAPK, focal adhesion kinase (FAK)-PI3K, and RhoA/ROCK pathways. These collectively contribute to enhanced invasiveness, metastasis, and angiogenesis (23). In human BLCA, CD147 upregulates gasdermin D (GSDMD), a key effector of pyroptotic cell death, thereby promoting tumor cell proliferation and influencing inflammatory caspase activity (57, 58).

Several additional signaling pathways regulated by CD147, including Wnt/β-catenin and TGF-β, have been shown to play major roles in tumor growth and metastasis. One of the CD147-mediated signaling cascades, the Wnt/β-catenin pathway, functions in TME modulation (59, 60) and within cancer cells themselves (61), thereby accelerating tumorigenesis (62). Elevated CD147 expression in lung cancer epithelial cells activates the Wnt/β-catenin pathway, whereas silencing CD147 suppresses tumor growth in xenograft mouse models (62). Through this pathway, CD147 promotes EMT-driven metastasis in prostate cancer by disrupting the β-catenin/E-cadherin complex, resulting in β-catenin nuclear translocation, activation of EMT-related genes, and E-cadherin loss (61). In addition, increased CD147 expression on soluble EVs positively correlates with Wnt/β-catenin activation in recipient CAFs, which subsequently stimulates the PI3K/Akt pathway and induces HIF-1 expression (59). Downstream Wnt/β-catenin target genes such as *c-Myc* and *HIF-1α* further reprogram cancer metabolism, promoting aerobic glycolysis and lactate export to the extracellular milieu (59)—a hallmark of Warburg effect that facilitates tumor growth and metastasis (29, 30). Moreover, CD147 supports Wnt signaling in maintaining T-cell lineage commitment, as evidenced by the downregulation of lineage-associated genes (*Notch1*, *Hes1*, *Dtx1*) and transcription factors (*Ets1*, *Gata3*, *Tcf1*) in CD147-deficient thymocytes from mice (60). TGF-β signaling represents another key axis through which CD147 exerts its oncogenic influence (63, 64). This pathway mediates proliferation and migration of endothelial cells (65) and drives EMT in several malignancies. In human HCC, CD147 promotes hepatocyte EMT through TGF-β–dependent upregulation of the transcription factor SLUG (63). *In vitro* experiments using HCC cell lines (SMMC-7721 and HepG2) demonstrated that CD147 overexpression correlates with increased SLUG and SNAIL, whereas CD147 silencing decreases N-cadherin, vimentin, and colony formation ability (63). Similarly, in tongue squamous cell carcinoma (SAS) cells, TGF-β treatment upregulates CD147 and vimentin, downregulates E-cadherin, and enhances cancer cell migration (64). A positive feedback loop between CD147 and TGF-β has been documented across multiple contexts, including liver fibrosis (66), HCC plasticity (67), and lung PMN formation (65). In the liver, overexpressed CD147 enhances the migratory capacity of hepatic stellate cells and potentiates TGF-β1–induced contraction (66). CD147 also sustains HCC malignancy by upregulating TGF-β1 transcription via β-catenin activation (67). Furthermore, CD147-induced MMP production facilitates the conversion of pro-TGF-β1 into its active form, thereby amplifying dedifferentiation processes that drive tumor progression (67). In a lung PMN mouse model, elevated CD147 levels from primary tumors increase TGF-β secretion, together with VEGF and MMP-9, promoting angiogenesis, fibroblast activation, neutrophil infiltration, and ECM remodeling (65).

Among the tumor-derived molecules that mediate PMN formation, the S100A8/A9-CD147 axis has emerged as a critical regulator. S100A8/A9, an inflammatory heterodimer, binds to CD147 and activates downstream signaling cascades such as ERK and NF-κB, facilitating tumor progression in HNSCC, melanoma, and HPV-positive penile cancer (68, 69). This interaction enhances cancer cell invasiveness and contributes to immune suppression, thereby fostering a tumor-permissive environment at distant metastatic sites. Exosomes and microvesicles mediate long-distance intercellular communication during metastasis, where these vesicles carry diverse bioactive cargos—proteins, lipids, metabolites, and nucleic acids (e.g., mRNAs, microRNAs, circular RNAs)—which are selectively loaded and secreted by tumor cells. Through these contents, EVs influence immune cell function (e.g., impairing NK cells and dendritic cells), induce EMT, and promote stromal remodeling and angiogenesis in target organs (6). Exosomal integrins, for instance, determine the organotropism of metastasis by directing vesicle uptake to specific tissues, while miR-934 and miR-519a-3p modulate macrophage polarization and neovascularization. In colon cancer stem cells, CD147 promotes EV release during differentiation, enhances tyrosine phosphorylation signaling in recipient cells, and contributes to increased invasiveness and EV uptake efficiency (70). Interestingly, CD147 has been shown to identify a distinct subset of EVs that are rich in miRNAs and primarily derived from cancer cells. These CD147^+^ EVs differ from classical tetraspanin^+^ EVs and are characterized by selective miRNA enrichment through interactions with heterogeneous nuclear ribonucleoprotein A2/B1 (HNRNPA2/B1) in human cell lines. Elevated levels of CD147^+^ EVs have been detected in the plasma of patients with ovarian and renal cancers, suggesting their potential as diagnostic and prognostic biomarkers (71). Post-translational modifications of CD147 further modulate its functional roles in cancer and EV-mediated metastasis. In breast cancer, high-mannose and complex-type N-glycosylation at specific residues (Asn-160 and Asn-268) of CD147 enhances the invasive potential of EVs and facilitates matrix degradation (72). Conversely, di-methylation of CD147 at Lys-71 (K71me2), catalyzed by the methyltransferase SETDB1, appears to inhibit tumor progression in NSCLC by promoting apoptosis via p38 MAPK signaling (73). These findings highlight the dual regulatory nature of CD147 modifications, which can either enhance or suppress its pro-tumorigenic activity depending on the cellular context.

Taken together, the evidence highlights the structural and functional versatility of CD147 in cancer biology. Its unique domain architecture enables diverse protein–protein interactions and regulates both intracellular signaling and extracellular communication, positioning it as a key driver of tumor progression and metastasis. By promoting immune evasion, metabolic adaptation, and PMN formation—particularly through modulation of tumor-derived EVs—CD147 plays a central role in the metastatic cascade. Given its multifaceted influence across several cancer hallmarks, CD147 represents a promising therapeutic target with potential applications in early detection, prognostication, and treatment of metastatic disease.

## 4 CD147-driven metabolic reprogramming: mechanisms underlying tumor progression and therapeutic resistance

**Figure 1:**
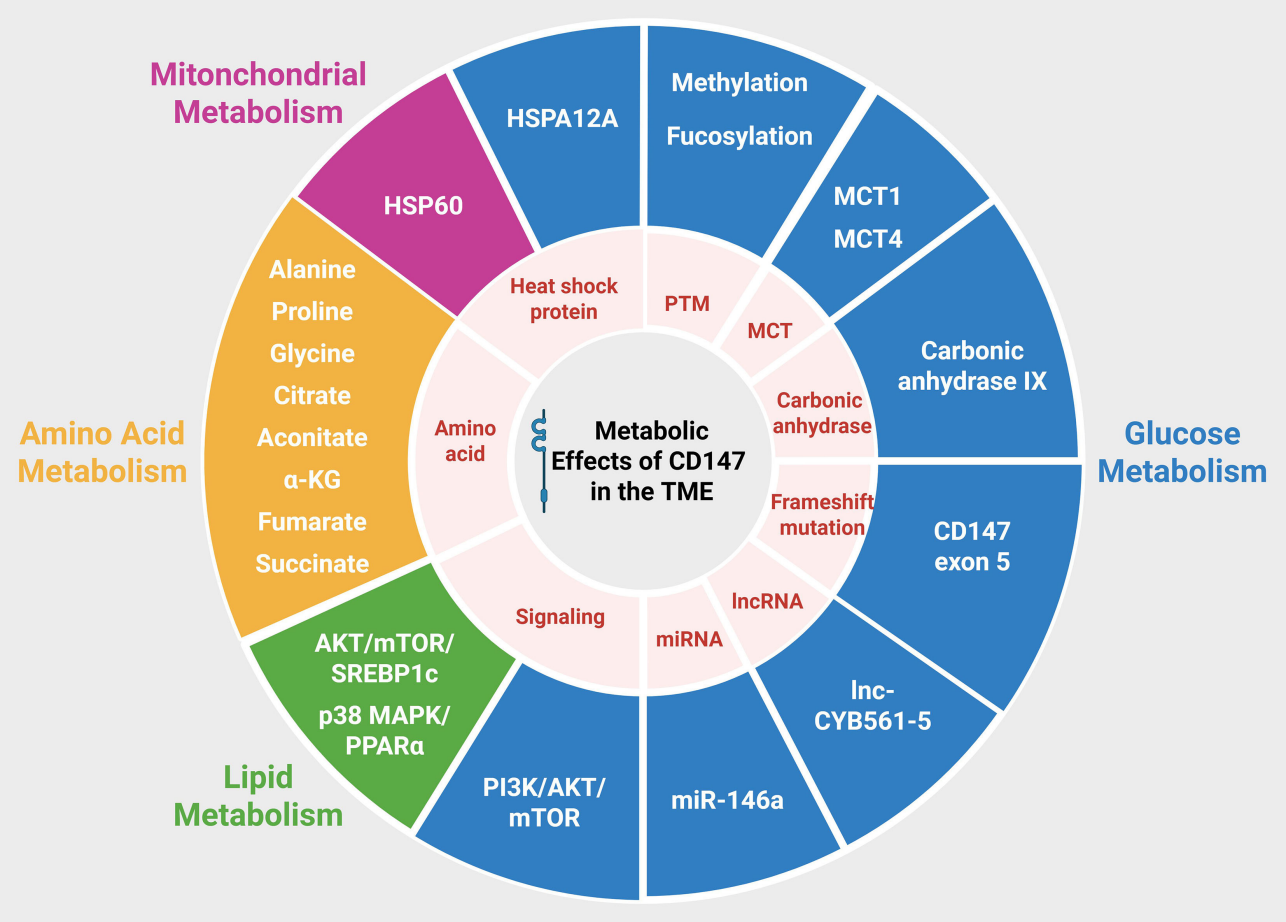
Overview of CD147-mediated metabolic reprogramming within the TME. CD147 modulates glucose, lipid, amino acid, and mitochondrial metabolism through multiple mechanisms across various cancer types. Substantial evidence indicates that CD147 predominantly influences glucose metabolism. PTMs of CD147, including methylation and fucosylation, together with its interaction with MCT1 and MCT4, facilitate lactate export and enhance glucose utilization. Overexpression of CD147 activates the PI3K/AKT/mTOR signaling cascade, leading to increased glucose uptake and elevated lactate production. In addition, CD147 interacts with the long non-coding RNA lnc-CYB561-5, which modulates the expression of glucose metabolism–related genes such as PFK1, G6PI, PCK2, and HK2. CD147 also regulates miR-146a–mediated aerobic glycolysis. Conversely, disruption of the CD147–MCT–carbonic anhydrase IX complex or frameshift mutations in exon 5 of CD147 reduce glycolytic activity and lactate secretion. Through its interactions with various HSPs, CD147 can influence both glucose and mitochondrial metabolism. In lipid metabolism, CD147 activates the AKT/mTOR/SREBP1c and p38 MAPK/PPARα signaling pathways, thereby modulating lipogenesis and fatty acid oxidation. Regarding amino acid metabolism, CD147 reshapes the TME to favor the availability of specific amino acids; CD147 depletion increases the intracellular levels of alanine, proline, glycine, citrate, aconitate, and α-KG, while reducing those of succinate and fumarate.

CD147, recognized for its role in cancer metabolism (74), functions as a pivotal modulator in cancer progression (28, 68, 75, 76) and chemoresistance (77), wherein metabolic alterations driven by CD147 contribute to oncogenic phenotypes and the establishment of PMN. In NSCLC, CD147-K234me2—a di-methylated variant of CD147 at Lys-234 catalyzed by lysine methyltransferase 5A (KMT5A)—enhances glycolytic flux and lactate export, thereby promoting tumor progression (78). Loss of KMT5A or expression of a CD147 lysine mutant (K234R) reduced glucose uptake, LDH activity, and lactate secretion in A549 and H460 cells. *In vivo*, tumors harboring the CD147-K234R mutation exhibited reduced volume and weight relative to wild-type CD147, with lactate levels emerging as a distinguishing metabolic marker among the 15 monitored metabolites. These findings underscore a mechanistic link between CD147-K234 methylation, enhanced glycolysis, and NSCLC progression (78). Lactate export is also mediated via the CD147/MCT4 complex. Treatment with the anti-tumor compound Formosanin C in NSCLC cells resulted in downregulation of MCT4 and CD147, intracellular lactate accumulation, and impaired lactate efflux, accompanied by elevated glucose and pyruvate levels (79). Li et al. further demonstrated that Formosanin C disrupted mitochondrial homeostasis through increased ROS and mitochondrial peroxide production, supporting the hypothesis that lactate accumulation facilitates a metabolic shift from aerobic glycolysis to OXPHOS, ultimately inducing cell death (79–81). These results suggest that the CD147-MCT4 axis not only regulates energy metabolism but also modulates mitochondrial dynamics. Additionally, CD147 interacts with lnc-CYB561-5, which influences NSCLC prognosis, cellular proliferation, apoptosis, metastasis, and glycolysis (82). In human cell lines, knockdown of lnc-CYB561–5 initially upregulated *PFK1*, *G6PI*, *PCK2*, and *HK2* genes, likely as a compensatory response. However, metabolic flux analysis revealed increased oxygen consumption rate (OCR) and decreased extracellular acidification rate (ECAR), yielding a higher OCR/ECAR ratio—indicative of a metabolic shift toward OXPHOS. Overexpression of CD147 rescued ECAR and glycolytic activity in lnc-CYB561-5-deficient cells, in which downregulation of PFK1 and HK2 can be observed (82). The mechanisms of CD147-driven metabolic reprogramming are summarized in (**Table 1**) and illustrated in (**Figure 1**).

**Table 1:** Metabolic effects of CD147 in the tumor microenvironment.

Metabolic effects	Cancer types	Cell line	References
Glucose metabolism			
K234me2: ↑ lactate export	NSCLC	A549 and H460	(78)
accompanied with MCT4 to facilitate lactate export	NSCLC	H1299	(79)
mediate the upregulation of Hk2/Pfk1: ↑ glycolysis	NSCLC	H1299 and A549	(82)
cooperate with MCT1/4: ↑ glucose metabolism	LUAD	HCC827, H1975, PC9, and A549	(84)
disruption of MCT1/4-CAIX lactate transport: ↓ glycolysis	BC	MCF-7 and MDA-MB-231	(88)
α- (1, 2)-fucosylation: ↑ AKT/mTOR/4EBP1	HCC	Huh7, CLC13, 293T and 293FT	(90)
overexpression: ↑ PI3K/Akt/mTOR pathway, ↑ glucose uptake, ↑ lactate production	HCC	SMMC-7721and HepG2	(91)
essential for PI3K/AKT/mTOR axis activation and HIF-1α expression: ↑ glycolysis	CRC	HCT15 and LoVo	(77)
exon 5 mutation: ↓ lactate secretion	CRC	HCT116	(93)
promoted degradation: ↓ glycolysis, ↓ lactate export	RCC	Caki-1 and 786O	(94)
Lipid metabolism			
silencing: ↓ FASN, ACOX1, ↓ lipid metabolism	LUAD	A549 and H1299	(83)
silencing: ↓ lipid content, ↓ Akt/mTOR/SREBP1c pathway, ↓ lipogenesis, ↑ fatty acid oxidation	HCC	SMMC-7721 and MHCC97L	(92)
silencing: ↑ PPARα, ↑ fatty acid oxidation	CRC	HCT15 and LoVo	(77)
Amino acid metabolism			
depletion: ↑ glutaminolysis, alanine/proline/glycine biosynthesis	PDAC	L3.6pl, L3.5, HS-766T, BxPC3, PANC1, and MiaPaCa2	(74)
knockdown: ↑citrate, aconitate, α-KG; ↓ succinate, fumarate	ALCL	SUDHL-1, KiJK, Karpas 299, SUP-M2, SR-786, Mac-1, Mac2a, and FE-PD	(100)
Mitochondrial metabolism			
interaction with HSP60: ↑ ATP5B, mitochondrial respiration	melanoma	SK-MEL-5, SK-MEL-28, A375, G361, MM200, and 293T	(95)

↑: up/promote; ↓down/inhibit. ALCL, anaplastic large cell lymphoma; BC, breast cancer; CRC, colorectal cancer; HCC, Hepatocellular Carcinoma; LUAD, lung adenocarcinoma; NSCLC, non-small cell lung cancer; OSCC, oral squamous cell carcinoma; PDAC, pancreatic ductal adenocarcinoma; RCC, renal cell carcinoma

### 4.1 Lung adenocarcinoma

In LUAD, CD147 suppresses lipid metabolism (83) and serves as an imaging biomarker for ^18^F-FDG PET/CT in targeted therapy (84). CD147 knockdown in A549 and H1299 cells decreased *FASN* and *ACOX1* mRNA levels—genes integral to lipid metabolism (85–87). Mechanistically, CD147 silencing inhibited the Rap1 signaling pathway in human cell lines, while Rap1 activation reversed the metabolic suppression by restoring FASN and ACOX1 expression (83). PET imaging further revealed a marked association between CD147 expression and SUVmax, SUVmean, and SUVpeak, suggesting CD147-mediated enhancement of glycolytic activity. CD147 was also shown to functionally cooperate with MCT1 and MCT4, with their expression levels correlating with both PET readouts and CD147 abundance (84).

### 4.2 Breast cancer

In BC, MCT1 and MCT4 (88), alongside signaling mediators such as p38/MAPK (72) and MMP14 (89), play crucial roles in cancer metabolism. CD147-positive microvesicles were enriched in BC patients and associated with enhanced invasiveness (72). Tumor-derived vesicles displayed differential glycosylation of CD147, with highly glycosylated variants correlating with invasiveness, as determined via mass spectrometry. Notably, glycosylation sites at Asn-160 and Asn-268 were absent in the highly glycosylated isoform. While MMP2 and MMP9 were undetectable, p38 phosphorylation was abolished upon CD147 knockdown, indicating an MMP14-independent invasion mechanism (72). CD147 also interacts with carbonic anhydrase IX (CAIX) to form a metabolon with MCT1/4, facilitating proton-coupled lactate transport and acid-base regulation within the TME (88). The interaction, likely mediated via CD147-Glu73 and CAIX-His200, was disrupted by anti-CD147 antibodies targeting the Ig1 domain, impairing complex assembly, suppressing glycolysis, and reducing cell proliferation. Moreover, the MCT4–CD147–MMP14 complex localizes to invadopodia, where it regulates ECM degradation through MMP-dependent collagen I remodeling, reinforcing CD147’s role in metabolic adaptation and invasion (89).

### 4.3 Hepatocellular carcinoma

In HCC, glucose deprivation induces cancer stem-like traits via FUT1-mediated α- (1, 2)-fucosylation of CD147 (90), while CD147 also reprograms glucose and lipid metabolism to promote immunosuppression and metastasis (91, 92). Under glucose restriction, CD147 fucosylation activates the AKT/mTOR/4EBP1 axis, enhancing stemness and survival (90). CD147-overexpressing HCC cells displayed increased ^18^F-FDG uptake, LDH activity, lactate production, and medium acidification, via activating the PI3K/Akt/mTOR signaling pathway. These effects correlated with *in vivo* glucose uptake and FOXP3^+^ Treg infiltration, highlighting CD147’s role in fostering an immunosuppressive niche through lactate accumulation (91). CD147 regulates fatty acid metabolism by activating the Akt/mTOR/SREBP1c pathway, which upregulates ACC1 and FASN, while simultaneously inhibiting fatty acid oxidation through suppression of the p38/PPARα axis (92). CD147 knockdown led to decreased intracellular triglycerides, phospholipids, and key fatty acids (e.g., oleic, stearic, palmitoleic, palmitic acids) (92). Collectively, CD147 enhances tumor growth and metastatic potential in HCC by orchestrating lipid metabolic reprogramming through dual signaling pathways.

### 4.4 Colorectal cancer

In CRC, CD147 expression correlates with chemoresistance and poor prognosis (77, 93), where aberrant glycolipid metabolism mediated by CD147 has been identified as a contributing factor (77). Acquired resistance to 5-fluorouracil (5-FU) in CRC cell lines is associated with increased glucose uptake, enhanced lactate production and flux, and elevated intracellular triglyceride and cholesterol levels, alongside reduced oxygen consumption, mitochondrial respiration, lipid utilization, and fatty acid oxidation (77). Notably, CD147 knockdown restores OXPHOS-dominant metabolism through modulation of two key pathways: PI3K/AKT/mTOR/HIF-1α, which regulates glucose metabolism, and MAPK/PPARα, which controls lipid metabolism (77). CD147 enhances glycolysis through activation of the PI3K/AKT/mTOR pathway and upregulation of HIF-1α, while concurrently inhibiting fatty acid oxidation via activation of the MAPK pathway and downregulation of PPARα. In a xenograft mouse model established from a 5-FU-resistant rectal cancer patient, combined treatment with 5-FU and a CD147 inhibitor resulted in significantly greater suppression of tumor growth, cell proliferation, and glycolipid metabolism than 5-FU monotherapy, highlighting CD147’s role in mediating 5-FU resistance in colorectal cancer by orchestrating glycolipid metabolic reprogramming (77). Identified in the context of the novel ruthenium-based anticancer agent BOLD-100 (sodium trans-[tetrachlorobis(1H-indazole)ruthenate(III)]), CD147 contributes to drug resistance through its interaction with MCT1 (93). Despite enhanced glycolytic activity in BOLD-100-resistant CRC cells, a frameshift mutation in exon 5 of the CD147 gene leads to the loss of MCT1, resulting in reduced lactate secretion (93). The premature translational stop caused by this mutation disrupts the transmembrane and cytoplasmic domains of CD147, which are essential for its interaction with and stabilization of MCT1 at the cell membrane (93). These findings underscore the role of CD147 in regulating cancer metabolism under drug-resistant conditions.

### 4.5 Pancreatic ductal adenocarcinoma

In PDAC, CD147 knockdown induces substantial proteomic changes, leading to metabolic reprogramming and the promotion of mesenchymal-epithelial transition (MET) (74). Alterations in metabolic enzyme expression were observed following CD147 depletion in human pancreatic cancer cell lines, including downregulation of G6PD and PKM2, alongside upregulation of phosphoglycerate dehydrogenase (PHGDH), GLUT1, pyrroline-5-carboxylate reductase 1 (PYCR1), hexokinase domain containing 1 (HKDC1), and ornithine aminotransferase (OAT) (74). The expression of transglutaminase 2 (TGM2) and aldehyde dehydrogenase 1A3 (ALDH1A3), both mesenchymal markers, reduce in CD147-deficient cells (74). Importantly, CD147 regulates amino acid metabolism, where impaired lactate export in CD147-silenced PDAC cells triggers glutaminolysis, leading to enhanced biosynthesis and secretion of amino acids including alanine, proline, and glycine. Elevated expression of alanine/serine/cysteine transporter 2 (ASCT2) and PHGDH further supports increased glutamine uptake and activation of the serine/glycine biosynthetic pathway, contributing to nucleotide biosynthesis and a modest increase in *de novo* DNA synthesis, reflecting a compensatory anabolic response to metabolic stress (74). Despite these metabolic adaptations, CD147-depleted cells experience a halt in cell cycle progression at the S/G2 phase (74), which in turn suppresses proliferation without triggering cell death—likely due to the inability to fully complete the cell cycle under metabolic constraint.

### 4.6 Renal cell carcinoma, melanoma and glioma

Metabolic reprogramming mediated by the CD147-heat shock protein (HSP) complex can be seen in RCC (94) and melanoma (95). In human RCC cell lines, CD147 is destabilized by HSPA12A (94), which is inversely associated with tumor metastasis. Overexpression of HSPA12A promotes CD147 ubiquitination and proteasomal degradation through its interaction with the E3 ubiquitin ligase, HMG-CoA reductase degradation protein 1 (HRD1), thereby reducing glycolysis, lactate export, extracellular acidification, and cell migration in 786O and Caki-1 cells (94). In metastatic melanoma, CD147 undergoes a redistribution from the plasma membrane to the cytoplasm and subsequently to the mitochondria during tumor progression (95, 96). The cytoplasmic form serves as an intermediate stage in this translocation process. Immunohistochemistry, immunofluorescence, immunoelectron microscopy, and mitochondrial fraction Western blotting confirmed the mitochondrial localization of CD147 in advanced melanoma (95). Although the precise molecular mechanism governing this translocation remains unclear (95), it is proposed to occur in response to metabolic stress or hypoxia (97, 98). Within mitochondria, CD147 interacts with HSP60, which in turn activates ATP5B, a subunit of complex V, thereby enhancing OXPHOS, ATP production, and ROS generation (95). This CD147–HSP60–ATP5B axis promotes mitochondrial aerobic respiration and correlates with increased invasive potential (95), suggesting that cytoplasmic and mitochondrial CD147 cooperatively drive metabolic reprogramming to support melanoma progression. In glioma, tumor progression is linked to metabolic reprogramming characterized by increased expression of CD147 and MCT1. The levels of CD147 and MCT1, along with HIF-1α, HK2, and LDH, are markedly upregulated in hypoxic glioma cells. Furthermore, elevated expression of CD147 and MCT1 is associated with increased exosome release in a calcium-dependent manner under hypoxia- or lactate-induced tumor microenvironmental conditions (99).

### 4.7 Anaplastic large cell lymphoma

In ALK^+^ ALCL, CD147–MCT1 complexes are essential for glucose metabolism and tumor growth, while CD147 knockdown significantly reduces glucose uptake and induces mitochondrial dysfunction. CD147, a target of miR-146a which differentially expressed between ALK^+^ and ALK^-^ ALCL, is downregulated upon miR-146a overexpression. In ALK^+^ subtypes, CD147 promotes tumor growth and invasion by enhancing miR-146a-mediated aerobic glycolysis. Alterations in nucleotide, amino acid, and lipid metabolism, along with the accumulation of lipids (triacylglycerols, lyso-phospholipids, and free fatty acids) and increased hexose levels are observed in CD147-depleted cells, possibly due to downstream blockade of glycolysis. Targeted TCA cycle analysis reveals increased levels of citrate, aconitate, and α-ketoglutarate (α-KG), an elevated α-KG/citrate ratio indicative of enhanced glutamine reductive carboxylation, and decreased levels of succinate and fumarate, suggesting compensation for mitochondrial dysfunction and impaired electron transport chain activity. Following mitochondrial damage, a metabolic shift is evident, as shown by higher basal and maximal OCRs in CD147-depleted cells compared to the control counterpart (100).

In summary, CD147 drives metabolic reprogramming across diverse cancer types by enhancing glycolysis, regulating amino acid and lipid metabolism, and promoting lactate export and glucose uptake. Through interactions with MCT1/4, lncRNAs, and key signaling pathways such as PI3K/AKT/mTOR, MAPK, and Rap1, CD147 supports tumor growth, invasion, and therapy resistance. Its influence on mitochondrial dynamics, stemness, and immune evasion further underscores its potential as a therapeutic target in cancer.

## 5 Coordinated roles of CD147 and CD44 in tumor progression and microenvironmental regulation

Interactions between CD147 and protein partners beyond MCTs indicate CD147’s central role in cancer metabolic reprogramming (74), a hallmark of PMN formation (6). This centrality is further underscored by CD147’s interactions with a variety of glycoproteins, including CD44 and other adhesion and signaling molecules, many of which are themselves key mediators of PMN development (101, 102). These glycoproteins contribute to ECM remodeling, immune cell modulation, and vesicle trafficking—processes fundamental to priming distant sites for metastatic colonization (103, 104). In the context of PMNs, CD44 plays a critical role by promoting EMT, increasing cell motility and invasion, and facilitating tumor–stroma interactions. This functional role of CD44 in PMNs provides a logical bridge to CD147, which cooperates with CD44 in several contexts. CD147 can form complexes with CD44 in lipid raft domains, enhancing hyaluronan–CD44-dependent EGFR-Ras-ERK signaling andinvadopodia formation, thereby promoting an invasive phenotype. Thus, their interaction synergistically contributes to PMN conditioning and metastatic colonization (105, 106). The functional consequences of CD147–CD44 networks are outlined in (**Table 2**).

**Table 2:** Coordinated roles of CD147 and membrane glycoproteins in cancer metastasis.

Coordinated roles	Cancer types	Cell line	References
CD44			
CD147-CD44-EGFR complex formation: ↑ invasiveness	BC	MDA-MB-231 and MCF-7	(119)
CD147-CD44 interaction regulates MCT membrane localization: ↑ glycolysis, ↑ efflux lactate		MCF-7, MDA-MB231, and MDA-MB436	(120)
CD147 increase membrane localization of CD44: ↑ stem-like behaviors		MDA MB231	(121)
CD147 and CD44 co-expressed cells: positive correlate with poor differentiation	OSCC	UPCI: SCC029B, AW13516, UPCI: SCC040, CAL-27, UPCI: SCC103, and UPCI: SCC16	(122)
CD147 and CD44 co-expression cell: ↑ ABCG2 and Bcl-2 expression		SCC-55	(123)
Increase membrane localization of CD44: ↑ stem-like behaviors	OAC	SKOV3	(121)
CD147 knockdown decreases CD44 expression: ↓ proliferation, ↓ metastasis, ↓ chemoresistance	PCa	PC-3M-luc-C6	(124)
EGFR			
Soluble CD147 promote EGFR-targeted IgA ADCC of neutrophil	HNSCC	CAL-33, FaDu, HN, HSC-4, SAS, SAT, UM-SCC-1, UPCI-SCC-154, and UPCI-SCC-090	(52)
Co-modified by fucosylation of CD147 and EGFR: ↑ cancer stemness	HCC	Huh7 and CLC13	(90)
CD147-CD44-EGFR complex formation: ↑ invasiveness	BC	MDA-MB-231 and MCF-7	(119)
CD147 promote EGFR initiated AKT/mTOR pathway: ↑ ^18^F-FDG uptake and glucose metabolism	NSCLC	HCC827 and H1975	(126)
CypA/CD147-EGFR axis: ↑ cancer stem cell growth		A549, NCI-H1299, NCI-H1650, and HCC827	(127)
Integrin			
CD147-Integrin αMβ2-Kindlin-3 axis: ↑ TAN polarization	HNSCC	CAL-33, FaDu, HN, HSC-4, SAS, SAT, UM-SCC-1, UPCI-SCC-154, and UPCI-SCC-090	(52)
CD147-Integrin αMβ2 axis: ↑ leukocyte and platelet adhesion	–	leukocyte and platelet cell	(13)
antibody block of CD147: ↓ α3/α6 integrins, ↓ FAK, ↑ caspase-3, ↑ JNK, ↑ p38 MAPK, ↑ SMAD pathways, ↑ tumor shrinkage	PDAC	PANC-1, MIA PaCa-2, and BxPC-3	(130)
	HCC	Hep G2	
	CML	KU812	
CD280 (MRC2)			
CD147 interacts with CTLD4 in MRC2: ↑ EMT	PCa	PC3 and DU145	(133)
	Melanoma	B16-F10	
CD276 (B7-H3)			
CD147-CD276 colocalization in lipid rafts: ↑ cancer stemness	BC	MDA-MB453	(137)

↑: up/promote; ↓down/inhibit. ALCL, anaplastic large cell lymphoma; BC, breast cancer; CML, chronic myeloid leukemia; HCC, Hepatocellular Carcinoma; HNSCC, head and neck squamous cell carcinoma; LUAD, lung adenocarcinoma; OAC: ovarian adeno-carcinoma; OSCC, oral squamous cell carcinoma; NSCLC, non-small cell lung cancer; PCa, prostate cancer; PDAC, Pancreatic Ductal Adenocarcinoma; PDAL, pancreatic ductal adenocarcinoma; RCC, renal cell carcinoma.

CD147 undergoes extensive post-translational modifications (PTMs) that critically influence its biological functions and interactions. These modifications—including methylation (73, 78, 107), fucosylation (90), phosphorylation (108), glycosylation (72, 109–112), acetylation (93), and ubiquitination (113) —primarily occur within or around the transmembrane domain, which serves as a key interface for protein–protein interactions (74, 114, 115). PTMs not only dictate the spatial conformation and stability of CD147 but also modulate its capacity to engage in multiprotein complexes within specialized plasma membrane microdomains, thereby enhancing its functional versatility. These biochemical alterations are strongly associated with aggressive cancer phenotypes, including enhanced metastatic potential, resistance to chemotherapeutic agents, and immune evasion. Mechanistically, PTM-mediated modulation of CD147 promotes metabolic reprogrammingas well as alterations in the TME through CD147–CD44 interactions. As a transmembrane glycoprotein implicated in cell adhesion, cytoskeletal remodeling, and signal transduction (106, 116), CD44 synergizes with CD147, particularly within hyaluronan-enriched microenvironments to coordinate downstream signaling pathways involved in tumor progression. Given the pivotal role of CD147 PTMs in malignancy, they are promising therapeutic targets, with monoclonal antibodies under investigation to disrupt pathological interactions and downstream effects (117, 118).

In breast cancer, interactions between CD147, CD44, hyaluronan, EGFR, and MCT regulate tumor invasiveness and lactate metabolism (119, 120). Recent *in vitro* investigations have elucidated that CD147 facilitates the assembly of a multiprotein complex comprising CD147, CD44, and EGFR in lipid raft-associated microdomains of the cell membrane, thereby enhancing the invasion of breast cancer cells such as MDA-MB-231 and MCF-7. This cooperative interaction is critically dependent on hyaluronan–CD44 binding, which in turn activates downstream EGFR-Ras-ERK signaling cascades. Specifically, the overexpression of CD147 has been shown to markedly increase Ras activation and subsequent phosphorylation of ERK1/2, both of which are attenuated upon pharmacological inhibition of EGFR, underscoring the upstream role of EGFR in this pathway. Moreover, CD147 overexpression induces a robust phosphorylation of the Tyr-1068 residue on the cytoplasmic tail of EGFR—a key docking site for adaptor proteins involved in Ras-MAPK signaling. Notably, silencing CD44 via siRNA leads to a substantial reduction in EGFR Tyr-1068 phosphorylation and ERK activation, implicating CD44 as an essential mediator in CD147-driven signal transduction. These findings are further supported by proximity ligation assays and co-immunoprecipitation experiments, which confirm the physical association among CD147, CD44, and EGFR within lipid raft domains. Intriguingly, Ras not only acts as a downstream effector but also positively regulates the expression of CD147 and hyaluronan synthesis, establishing a feed-forward loop that amplifies signal propagation and promotes cellular invasiveness. Collectively, these data highlight a synergistic interplay between CD147 and CD44 in orchestrating EGFR-dependent oncogenic signaling and underscore their promise as dual therapeutic targets in invasive breast cancer (119). Beyond its role in modulating EGFR-dependent signaling pathways, the CD147–CD44 complex also exerts significant influence over localization and stabilization of metabolic transporters that sustain tumor aggressiveness (120). Although CD147 forms complexes with CD44 at the plasma membrane, its membrane localization does not appear to be solely dependent on interactions with CD44 or hyaluronan (120). The disruption of CD44–hyaluronan interactions using hyaluronan oligosaccharides leads to CD44’s rapid internalization and redistribution throughout the cytoplasm (120). However, CD147 remains predominantly localized at the cell surface under these conditions (120), indicating that its membrane retention is maintained independently of CD44–hyaluronan-mediated stabilization and may involve alternative anchoring mechanisms, such as interactions with other membrane proteins or specific glycosylation patterns. This differential behavior underscores the unique regulatory roles of CD147 in contrast to CD44 in maintaining plasma membrane complexes essential for the PMN formation. A study on cancer stem-like traits revealed that, in MDA-MB-231 breast cancer cells, subpopulations with high CD147 surface expression show enhanced membrane localization of CD44, EGFR, MCT4, ABCB1, and ABCG2, highlighting their coordinated interactions (121). This membrane enrichment is functionally significant, as CD147 was shown to facilitate the assembly of multi-protein complexes that enhance oncogenic behaviors (121). Specifically, CD147 cooperates with CD44 to promote stem-like properties such as anchorage-independent growth, invasive capacity, chemoresistance, and spheroid formation (121). Simultaneously, its association with ABC transporters contributes to heightened drug efflux activity, thereby conferring a substantial survival advantage under chemotherapeutic stress, particularly against doxorubicin (121). Notably, these molecular features were observed not only in breast carcinoma but also in ovarian cancer, suggesting a broader relevance of CD147-associated membrane complexes in multiple tumor types (121).In oral squamous cell carcinoma (OSCC), the co-expression of CD147 and CD44 has been shown to mark a subpopulation of cancer cells with cancer stem cell-like traits, a phenotype that has also been observed in other malignancies such as breast cancer (122, 123). Specifically, in the SCC-55 cell line, a subset of cells identified as side population (SP) cells—characterized by Hoechst 33342 dye efflux via ABC transporters—exhibited elevated expression of both CD44 and CD147, highlighting their enrichment in cancer stem cell-like traits (123). This co-expression coincided with heightened resistance to chemotherapeutic agents such as 5-FU, attributed to the upregulation of ABCG2 and anti-apoptotic Bcl-2 proteins (123). Mechanistically, CD44 and CD147 cooperate to sustain side population (SP) cell survival and plasticity by activating signaling pathways that converge on EMT regulators and drug resistance genes (123). Their interaction may facilitate a positive feedback loop that not only stabilizes the mesenchymal phenotype but also enhances the expression of multidrug resistance transporters, thereby promoting tumor persistence, metastatic spread, and recurrence. Building on these findings, further evidence shows that CD44^+^/CD147^+^ cells exhibit enhanced self-renewal, migratory, and invasive capabilities, strongly associated with the upregulation of key EMT markers—including Vimentin, Snail, and Collagen 3A1—indicative of a mesenchymal, invasive phenotype (122). The CD147–CD44 interaction appears to synergistically promote tumor aggressiveness by elevating the expression of invasion-associated molecules such as MMP2, MMP9, and N-cadherin, while concurrently downregulating E-cadherin, a hallmark of epithelial integrity (122). Importantly, clinical analyses reveal that poor prognosis, including increased recurrence and reduced overall survival, correlates significantly with high CD147 and CD44 co-expression, but not with their individual expression (122).

In prostate cancer, knockdown of CD147 or CD44 reduces MCT4 and MRP2 expression, suppresses proliferation and invasion, and increases docetaxel sensitivity *in vitro* and *in vivo* (124). These effects are linked to decreased p-Akt and p-Erk levels, while CD44 and CD147 activation promotes PI3K/Akt and MAPK/Erk signaling, metastasis, and chemoresistance in PC-3M-luc-C6 cells (124). In the evolving landscape of PMN formation, the regulation of surface molecules such as CD147 and CD44 through PTMs plays a pivotal role in determining whether these glycoproteins are recycled back to the plasma membrane or targeted for degradation. These trafficking decisions directly influence cell adhesion, ECM remodeling, and intercellular communication—hallmarks of PMN dynamics. In a study utilizing HeLa cells, Eyster et al. demonstrated that membrane-associated RING-CH 8 (MARCH8), an E3 ubiquitin ligase, specifically ubiquitinates CD44, redirecting it from tubular recycling endosomes to early endosome antigen 1 (EEA1)-positive sorting endosomes and subsequently to lysosome-associated membrane protein 1 (Lamp1)-positive late-stage endosomes for lysosomal degradation (125). This rerouting is dependent on the engagement of the ESCRT apparatus, as knockdown of TSG101, a critical ESCRT-I component, effectively inhibits MARCH8-induced CD44 trafficking to the degradative pathway (125). Interestingly, CD147, which shares similar clathrin-independent endocytic routes with CD44 under basal conditions, remains largely unaffected by MARCH8 overexpression (125). This resistance may stem from differences in their cytoplasmic tail sequences and a relative lack of lysine residues in CD147, rendering it a poor substrate for ubiquitination (125). These findings suggest that selective ubiquitination mediated by MARCH8, coupled with ESCRT-dependent sorting, serves as a molecular switch governing the intracellular fate of key surface proteins, thereby potentially modulating the metastatic competence of the TME.

The CD147–CD44 axis, influenced by tumor microenvironmental factors, acts as a key regulator of tumor differentiation, invasion, and therapeutic resistance in various cancers. By forming a dynamic signaling hub with partners like EGFR and ABC transporters, it drives oncogenic pathways, metabolic adaptation, and drug resistance. Their molecular interplay modulates cell plasticity and metastasis, making the CD147–CD44 axis a promising therapeutic target to disrupt tumor aggressiveness and stem-like traits across malignancies.

## 6 Functional interplay between CD147 and membrane glycoproteins in the tumor microenvironment

**Figure 2:**
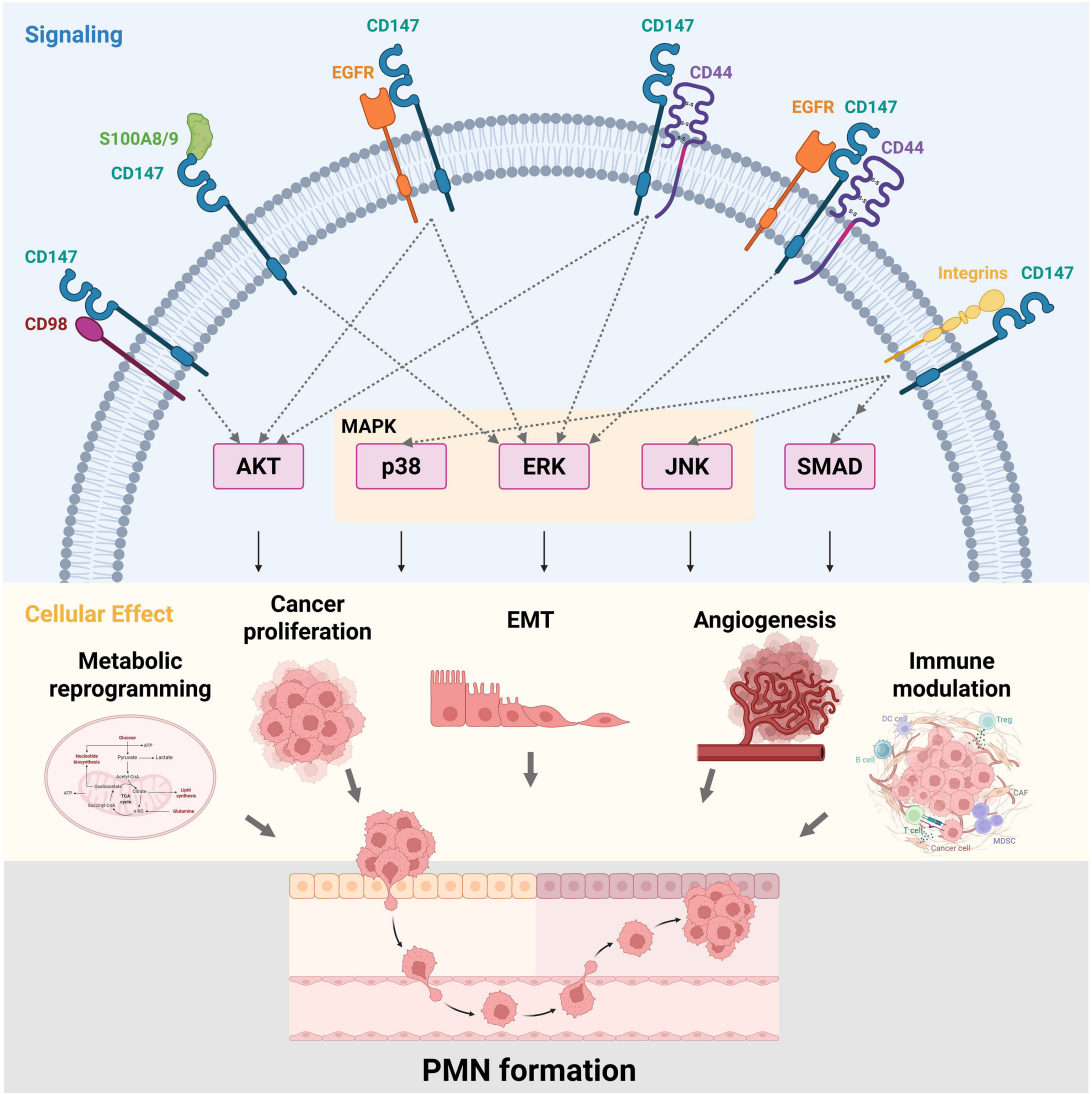
Overview of CD147-mediated protein interactions, signaling pathways, and functional outcomes. CD147 engages in diverse protein–protein interactions with partners such as CD98, S100A8/A9, EGFR, CD44, and integrins, thereby activating multiple downstream signaling cascades, including AKT, p38, ERK, JNK, and SMAD. The major pathways converge within the MAPK signaling network, wherein p38, ERK, and JNK represent distinct branches. CD147, in complex with EGFR or/and CD44, stimulates the AKT and ERK pathways. Whereas CD147-CD98 complexes predominantly stimulate the AKT pathway, and CD147-S100A8/A9 facilitates the ERK pathway. Interaction with integrins preferentially activates p38, JNK, and SMAD signaling. Through these coordinated signaling events, CD147-associated multiprotein complexes orchestrate metabolic reprogramming, tumor proliferation, EMT, angiogenesis, and immune modulation, collectively fostering a permissive niche for PMN formation.

The convergence of multiple glycoproteins interacting with CD147 leads to cancer stemness, antibody-dependent cell-mediated cytotoxicity (ADCC), EMT, and cell cycle arrest. Among these, EGFR, integrins, CD280, and CD276 have been widely reported. These glycoproteins, often embedded in or associated with lipid rafts and enriched in N- and O-linked glycosylation sites, share structural features that facilitate dynamic interactions with CD147’s extracellular Ig-like domains. These interactions are frequently glycosylation-dependent, influencing cellular signaling platforms, cytoskeletal rearrangements, and membrane localization, which are essential for maintaining tumor-promoting phenotypes such as immune evasion, motility, and therapeutic resistance. The functional consequences of CD147–glycoprotein networks are outlined in (**Table 2**) and illustrated in (**Figure 2**).

### 6.1 EGFR

Although CD147 does not directly interact with EGFR, its expression and secretion significantly influence the therapeutic efficacy of EGFR-targeted IgA antibodies by modulating neutrophil function in HNSCC (52). Soluble CD147 (sCD147) enhances IgA-mediated ADCC by promoting neutrophil activation in a dose-dependent manner, whereas membranous CD147 (mCD147) on neutrophils impedes effective IgA binding to its Fc receptor (FcαRI), thereby attenuating ADCC responses (52). In HPV^+^ HNSCC cell lines, elevated levels of sCD147 and reduced induction of mCD147 on neutrophils create a microenvironment more conducive to robust neutrophil-mediated cytotoxicity (52). This interplay between sCD147 and mCD147 modulates neutrophil responsiveness and determines tumor susceptibility to IgA-dependent killing. Furthermore, the functional interactions among CD147, Mac1, and Kindlin-3 influence FcαRI clustering, immune synapse formation, and downstream signaling (52). Within the PMN, this CD147–Mac1–Kindlin-3 axis supports neutrophil recruitment and activation, reshapes immune synapse architecture, and drives cytoskeletal reorganization—collectively promoting the transition toward tumor-associated neutrophil (TAN) phenotypes that facilitate metastatic progression (52). In HCC, CD147 and EGFR were identified as α- (1, 2)-fucosylated targets of FUT1, contributing to enhanced cancer stemness via activating the AKT/mTOR/4EBP1 pathway (90). Notably, prior studies have shown that the highly glycosylated form of CD147 interacts more robustly with EGFR, thereby promoting Ras/MAPK signaling (119, 126), supporting the idea that fucosylation of N-linked glycoproteins enhances their interaction potential and oncogenic signaling activity.

In NSCLC, CD147 is more highly expressed in EGFR-TKI-sensitive cell lines and promotes ^18^F-FDG uptake via an EGFR-Akt/mTOR pathway. CD147 also correlates with PD-L1 expression, which is reduced when Akt/mTOR is inhibited. These results support combining CD147-targeted therapy with EGFR-TKIs and anti-PD-1/PD-L1 immunotherapy for improved treatment (126). Additional promising anti-cancer agents include natural cyclophilin A (CypA) inhibitors, such as 23-demethyl 8,13-deoxynargenicin (C9) and cyclosporin A (CsA) (127). These compounds suppress self-renewal capacity, inhibit cell proliferation via activation of the intrinsic apoptotic pathway, and downregulate the CypA/CD147 axis as well as EGFR signaling in NSCLC cancer stem cells (CSCs). A dual therapeutic approach combining afatinib with either C9 or CsA has demonstrated a more robust inhibition of the CypA/CD147/EGFR axis, resulting in enhanced suppression of cancer cell proliferation and tumorsphere formation (127).

### 6.2 Integrin

Integrins, which are heterodimeric transmembrane receptors composed of one α and one β subunit, rely on proper functional regulation to mediate cell adhesion and signaling. Interactions between CD147 and integrins play a critical role in modulating cellular architecture (128), with integrin Mac-1 (αMβ2) recognized for its capacity to bind a wide array of ligands via its αMI domain (129). The direct, dose-dependent binding between CD147 and Mac-1 influences TAN polarization and facilitates leukocyte and platelet adhesion, as evidenced by significantly reduced adhesion in leukocytes from CD147^+^/^−^ and Mac-1^−^/^−^ mice compared to wild-type controls (13). By promoting platelet-leukocyte aggregation, adhesion, and pro-inflammatory signaling, the CD147–Mac-1 axis may contribute to PMN formation via recruiting immune cells and platelets that remodel the local vasculature or secrete pro-metastatic factors. In various malignancies, including PDAC, HCC, and chronic myeloid leukemia (CML), the function of integrins such as α3β1 and α6β1 is modulated by CD147 (130). CD147 facilitates the stabilization and recycling of these integrins on the cell surface by forming direct interactions with them, thereby maintaining their functional integrity. Targeted downregulation of CD147 using the monoclonal antibody h4#147D reduces surface expression of integrins α3 and α6, thereby destabilizing downstream signaling and leading to FAK inhibition and caspase-3 activation through stress pathways such as JNK, p38MAPK, and SMAD (130). The convergence of these signaling disruptions ultimately triggers apoptotic cell death, highlighting the critical role of CD147 in integrin-mediated oncogenic signaling.

### 6.3 uPARAP/Endo180

uPARAP/Endo180, also known as mannose receptor C-type 2 (MRC2) or CD280, is overexpressed in various cancers and functions as a recycling endocytic receptor that facilitates cell migration and the uptake of collagens for intracellular degradation (131, 132). MRC2 interacts with the highly glycosylated form of CD147 and VEGF receptors primarily via its C-type lectin-like domain 4 (CTLD4) (131, 133). Importantly, disruption of the CD147-MRC2 complex has been linked to the induction of EMT (133). In prostate cancer, modulation of CTLD4 alters CD147 glycosylation, driving its internalization and the disassembly of adherens junctions, thereby reinforcing the functional association between CD147 and MRC2 (133). This interaction plays a critical role in shaping the PMN by facilitating ECM remodeling, enhancing tumor cell motility, and promoting the recruitment of stromal and immune components that support metastatic colonization.

### 6.4 CD276

CD276 (B7-H3), a member of the B7 family of immune checkpoint molecules, is a glycoprotein that interacts with CD147 and plays a critical role in modulating the TME, promoting immune evasion, and contributing to therapeutic resistance (134–136). Depending on the receptor it engages, the B7 family can deliver either costimulatory or coinhibitory signals; B7-H3 predominantly acts as a negative regulator of immune responses and is frequently overexpressed in a variety of human cancers (134). In breast cancer, the colocalization of CD276 and CD147 within lipid rafts has been implicated in the maintenance of cancer stem cell properties. CD147-knockout cancer stem cells treated with docetaxel alone or in combination with methyl-β-cyclodextrin (MbCD) show increased p53 expression and decreased cyclin A levels, indicating that docetaxel-induced cell cycle arrest relies on proteins closely associated with CD147 (137).

Taken together, the broad spectrum of glycoprotein interactions with CD147 underscores its role as a central scaffold and signaling modulator within the TME. By forming stable or transient complexes with EGFR, integrins, MRC2, and CD276, CD147 coordinates diverse oncogenic pathways that drive stemness, EMT, immune suppression, and metabolic reprogramming. Among which the MAPK and AKT pathways play predominant roles. Activation of the MAPK pathway—including p38, ERK, and JNK branches—mediates cellular stress responses, metabolic adaptation, and tumor progression, collectively shaping a permissive microenvironment for metastasis. Concurrently, CD147-induced activation of the AKT pathway enhances cancer cell survival, proliferation, and stemness, thereby promoting malignancy and dissemination. Notably, these interactions are highly context-dependent and frequently modulated by PTMs, including glycosylation and fucosylation, which alter binding affinities and downstream signaling outcomes. As such, the CD147 interactome represents a compelling therapeutic target, where disrupting key glycoprotein associations may simultaneously attenuate multiple cancer hallmarks, providing a rationale for combinatorial strategies in precision oncology.

## 7 Advances in CD147-targeted therapeutic approaches

Previous research has focused on targeting PTMs of CD147, identifying bioactive compounds, and disrupting CD147-mediated EV secretion that contributes to PMN formation. Inhibiting CD147 phosphorylation and disrupting the Fyn–CD147 axis using amodiaquine effectively suppresses melanoma growth and metastasis (108). Blocking CD147 glycosylation with the synthetic compound methyl 3’-(4-chlorophenyl)-4’,5’-dihydro-[3,5’-biisoxazole]-5-carboxylate reduces MMP production, attenuates metastasis, and promotes E-cadherin expression in HeLa cells (110). Myricetin, a natural flavonoid, acts as a potent CD147 inhibitor by promoting its degradation and enhancing cisplatin efficacy through the suppression of DNA repair pathway genes and FOXM1, thereby increasing genomic toxicity (138). CD147 can also be targeted through cancer-derived EVs; for instance, combination of CD147 inhibitor and tissue factor pathway inhibitor significantly mitigates the pro-invasive effects of these EVs (139). Recent studies identify CD147-positive EVs as promising biomarkers for detecting colorectal neoplasia, advanced adenocarcinoma, and early-stage CRC, outperforming the fecal immunochemical test (FIT) in identifying precancerous lesions and proximal neoplasia (140). In HCC, combining chemotherapy with an autophagy inhibitor counteracts CD147-induced autophagy and restores mTOR activity, thereby improving chemosensitivity (141).

CD147 has also emerged as a viable candidate for antibody-based and cellular therapies. Metuzumab, a chimeric anti-CD147 monoclonal antibody with Fc glycoform modification, enhances ADCC and inhibits tumor cell migration and invasion by suppressing the PI3K/Akt signaling pathway and insulin-like growth factor 1 (IGF-1) expression. *In vivo*, Metuzumab significantly reduces tumor growth and lymph node metastasis in esophageal cancer models (142). Mechanistically, Metuzumab binds CD147 via its Fab region to block downstream signaling, while its glycoengineered Fc region enhances affinity for activating FcγRIIIA and diminishes binding to inhibitory FcγRIIB, thereby promoting NK cell and macrophage-mediated cytotoxicity (142). The humanized single-chain variable fragment (HuScFvM6-1B9), originating from the murine antibody M6-1B9, specifically targets the domain 1 epitope of CD147, retaining immunoreactivity and inhibiting T-cell proliferation (143). Evidence from structural modeling demonstrated that HuScFvM6-1B9 interacts with CD147 through specific hydrogen bonds (Ser-52, Tyr-56, and Tyr-58) and van der Waals interactions (Ala-33 and Phe-95) within its heavy chain. This interaction forms a stable complex that sterically hinders the access of MMPs, integrins, and MCTs that normally bind to the domain 1 epitope of CD147. By occupying this functional site, HuScFvM6-1B9 disrupts the interactions between CD147 and its partner proteins, thereby suppressing MMP induction, ECM degradation, and tumor cell invasion (143).

A follow-up research utilized computational affinity maturation to designate a point mutation in the light chain of the HuScFvM6-1B9 antibody, where Asn-32 was mutated to Tyr-32, engineering a variant called HuScFvM6-1B9^L1:N32Y^ (118). This substitution made HuScFvM6-1B9^L1:N32Y^ exhibiting increased CD147 binding affinity by enhancing hydrophobic interactions and π–π stacking at the antibody–antigen interface, strengthening its contact with the domain 1 epitope of CD147 (118). Additionally, Nb 11-1, an anti-CD147 nanobody, shows high affinity and specificity for CD147 in laboratory and animal studies. When conjugated with doxorubicin to form DOX-11-1, this nanobody selectively targets CD147^+^ tumor cells, inhibits growth, and induces apoptosis more effectively than free doxorubicin, while reducing toxicity to normal cells (144). Nb 11–1 is characterized by its binding to an epitope within domain 2 of CD147, in which both hydrophobic and hydrogen-bonding interactions contribute to complex stability. Specifically, a hydrophobic core is formed between Trp-98 of Nb 11–1 and Arg-300 of CD147, while hydrogen bonds are established between Arg-45 of Nb 11–1 and Asp-260 of CD147, and between Asn-102 of Nb 11–1 and Tyr-256 of CD147 (144).

**Figure 3:**
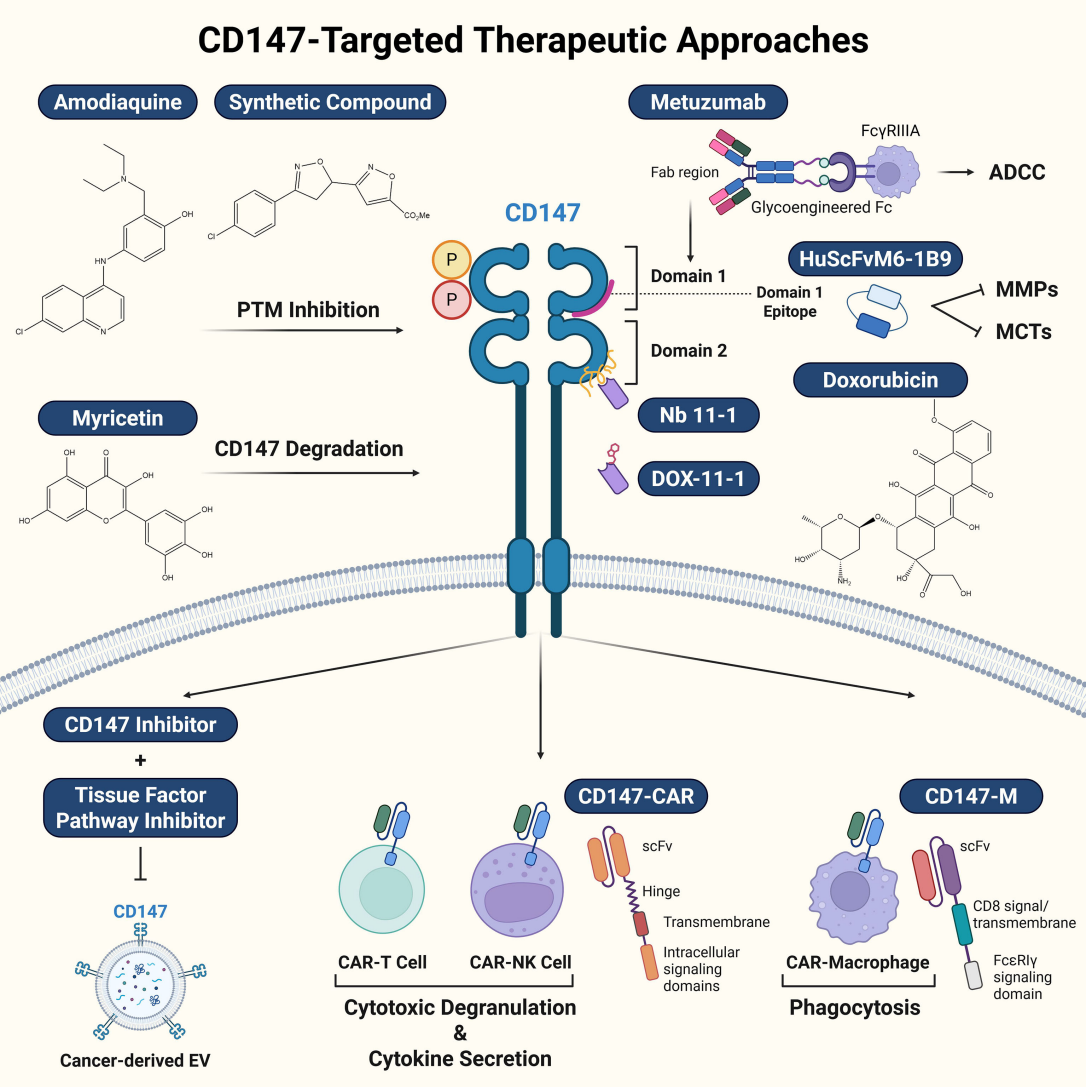
Overview of diverse therapeutic strategies targeting CD147 structure and signaling. Therapeutic interventions are categorized by their target mechanisms on the CD147 molecule—specifically PTM inhibition, antibody and nanobody targeting, and CAR therapy. PTM Inhibition: Small molecules target specific PTMs; amodiaquine inhibits CD147 phosphorylation, while synthetic compounds, such as 3’-(4-chlorophenyl)-4’,5’-dihydro-[3,5’-biisoxazole]-5-carboxylate, blocking glycosylation reduce MMP production. The flavonoid myricetin promotes CD147 degradation to suppress DNA repair pathways. Antibody and Nanobody Targeting: The glycoengineered antibody Metuzumab binds CD147 via its Fab region and enhances ADCC through a modified Fc region. The humanized single-chain variable fragment HuScFvM6-1B9 specifically targets the Domain 1 epitope, sterically hindering the access of MMPs and MCTs. In contrast, the nanobody Nb 11–1 binds to Domain 2 and can be conjugated with doxorubicin (DOX-11-1) for targeted drug delivery. CAR Therapy: Genetically engineered immune cells express CARs anchored in the cell membrane via transmembrane domains. CD147-CAR-T and CAR-NK cells utilize a CD28 transmembrane domain to trigger cytotoxic degranulation and cytokine secretion. CD147-CAR-Macrophages (CAR-M) incorporate a CD8 transmembrane domain and the FcεRIγ intracellular signaling domain to initiate phagocytosis of CD147-overexpressing tumor cells.

CD147-directed CAR-T and CAR-NK cells effectively eradicate HCC, and logic-gated systems improve tumor specificity and minimize off-tumor toxicity (145). The CD147-CAR construct comprises an anti-CD147 single-chain variable fragment (scFv), a human IgG1 hinge region, a CD28 transmembrane domain, and intracellular signaling domains that differ between T cells and NK cells. Upon binding of the scFv to CD147 on HCC cells, downstream activation cascades are triggered—mediated by CD28–4-1BB–CD3ζ signaling in T cells and by NKG2D-dependent pathways in NK cells—resulting in cytotoxic degranulation (CD107a expression) and the secretion of proinflammatory cytokines (TNF-α and IFN-γ), which collectively induce apoptosis or lysis of CD147^+^ HCC cells (145). Furthermore, CD147-specific CAR-macrophages (CAR-M) exhibit robust anti-tumor activity while maintaining safety for normal cells, offering a promising platform for solid tumor immunotherapy (146). Unlike the CAR constructs that used in previous studies, the CAR-M construct in this study incorporated a CD8 signal peptide, a CD8 transmembrane domain, and a human FcϵRIγ intracellular signaling domain, in addition to the anti-CD147 scFv. Upon binding of the scFv to CD147-overexpressing cancer cells, the immunoreceptor tyrosine-based activation motifs (ITAMs) within FcϵRIγ become phosphorylated, thereby initiating the phagocytosis of CD147^+^ tumor cells (146). The overview of diverse therapeutic strategies targeting CD147 structure and signaling is illustrated in (**Figure 3**).

## 8 Discussion

CD147 contributes to PMN formation by regulating metabolic reprogramming and ECM remodeling, while also promoting tumor invasion and metastasis through specific protein–protein interactions. It not only alters glucose, fatty acid, amino acid, and mitochondrial metabolism but also regulates stromal metabolism to support metastatic progression. In the TME, CD147-expressing small EVs activate CAFs, initiating β-catenin-mediated aerobic glycolysis and lactate release. This metabolic shift is accompanied by increased cytokine and VEGF secretion, driven by NF-κB and nitric oxide (NO) signaling, which collectively enhance cancer stem cell invasiveness and tumor growth (59). CD147 also promotes EMT through the disassembly of its protein complex with MRC2 (133), potentially leading to ECM degradation (147) and supporting cancer stemness (148). Conversely, CD147 contributes to ECM deposition through myofibroblast differentiation, emphasizing its dual role in both ECM degradation and reconstruction (14).

The unique structural conformation of CD147 enables it to function as a versatile signaling hub capable of homodimerization, oligomerization, and simultaneous association with multiple binding partners to form multiprotein complexes. For instance, CD147 monomers can dimerize and further self-associate depending on their glycosylation state and the extracellular binding of Zn(II) to the C-terminal domain, events that correlate with MMP induction (149). Beyond self-assembly, CD147 forms complexes on the plasma membrane with a range of partners, including MCTs, integrins, CD44, CD98, caveolin-1, and cyclophilins (16). These interactions are not mutually exclusive; for example, CD147–MCT complexes may coexist with CD44 or integrin assemblies, thereby coordinating metabolic, adhesive, and signaling processes (150).

The variability in CD147’s PTMs, membrane localization, trafficking dynamics, co-receptor expression profiles, and TME conditions across cancer types likely accounts for the distinct sets of CD147-associated complexes observed. This context dependency may explain why, despite CD147’s ubiquitous involvement, downstream signaling varies—such as enhanced PI3K/Akt activity in NSCLC (56), heightened MAPK/ERK sensitivity in BC (28), or Wnt/β-catenin predominance in certain epithelial malignancies (62). Acting as a multivalent scaffold rather than a simple monomeric receptor, CD147 orchestrates the selection of binding partners and thereby determines the activation of specific canonical signaling pathways. In addition to its canonical pathways, such as Wnt/β-catenin and TGF-β, CD147’s preference to primarily activate MAPK and AKT cascades when forming multiprotein complexes makes it an intriguing scaffold that may play a synergistic role among various signaling pathways, further driving tumor progression and metastasis.

Although CD147 represents a potential therapeutic target in metastatic cancer, its underlying mechanisms require further validation across diverse cancer subtypes. While CD147 has been identified as a valuable diagnostic indicator in multiple malignancies, its prognostic significance remains inconsistent. For instance, despite being overexpressed in esophageal cancer, one study reported that CD147 lacks predictive significance in both esophageal adenocarcinoma and esophageal squamous cell carcinoma (151), contradicting earlier findings (152). This discrepancy may be attributed to variations in sample size, tumor stage, and the use of neoadjuvant treatment. Consequently, further investigation is warranted to determine the prognostic significance of CD147 in distinct cancer subtypes. To address these challenges, the integration of artificial intelligence (AI) offers a powerful strategy through approaches such as integrative data analysis (153) and subtype-specific prognostic modeling (154). By combining multi-omic datasets, AI can identify robust gene expression markers predictive of drug sensitivity, as demonstrated in acute myeloid leukemia (AML) (153). Furthermore, machine learning algorithms have been employed to define prognostic subtypes based on TME characteristics, thereby improving survival prediction in NSCLC patients (154). Recent advances in AI-based protein interaction prediction, such as AlphaFold 3, further expand the potential for translational research. This diffusion-based deep learning model jointly predicts the structures of protein complexes, including those involving nucleic acids, small molecules, and ions. AlphaFold 3 significantly outperforms previous tools in modeling protein-ligand, protein-nucleic acid, and antibody-antigen interactions (155). Applying such technologies to CD147 could accelerate the discovery of novel interacting partners, enhance the precision of molecular docking, and facilitate the development of CD147-targeted antibodies. Ultimately, AI plays a pivotal role in addressing the limitations of current biomarker research by integrating and analyzing high-dimensional datasets to uncover meaningful biological patterns. It is increasingly used for target prediction, drug development, and immunotherapy response modeling—thus enhancing the clinical relevance and utility of biomarkers. Through these applications, AI transforms complex molecular data into actionable insights, advancing the personalization and effectiveness of cancer therapies (156, 157).

## 9 Conclusion

PMN formation is not a passive consequence of tumor progression but a highly orchestrated process driven by immune and stromal mediators, with CD147 positioned at its functional epicenter. As a master mediator, CD147 forms multiprotein complexes and integrates metabolic reprogramming, ECM remodeling, and intercellular signaling to facilitate metastatic colonization and progression. Its interactions with binding proteins—such as CD44, EGFR, integrins, CD280, and CD176—and its dual role in both tumor and stromal compartments highlight a unique therapeutic vulnerability that extends beyond conventional single-pathway targeting strategies.

Despite this promise, the clinical translation of CD147-targeted interventions remains hindered by tumor heterogeneity, context-dependent expression, and insufficient molecular stratification across cancer types. Emerging technologies— including AI-assisted multi-omic profiling, subtype-specific modeling, and protein—protein interaction—offer powerful opportunities to refine biomarker selection, clarify context-specific CD147 functions, and accelerate rational drug development. Moreover, dissecting the downstream signaling axes activated by CD147–protein interactions, such as the p38, ERK, JNK, AKT, and SMAD pathways, provides mechanistic insights that may guide tailored interventions for disrupting PMN formation.

Taken together, this review clarifies the integrative role of CD147 in orchestrating metastatic niche biology and highlights its potential as a targetable node within the interconnected immune and stromal networks that enable metastasis. Advancing CD147-directed precision therapeutics will require coordinated efforts to map context-specific interactomes, identify actionable biomarkers, and design agents that selectively disrupt its multiprotein complexes. By positioning CD147 at the center of new therapeutic frameworks, future studies may not only improve our understanding of metastatic progression but also drive the development of next-generation strategies to more effectively prevent and treat metastatic disease.

## Glossary

^18^F-FDG: ^18^F-fluorodeoxyglucose
5-FU: 5-fluorouracil
ABC transporter: ATP-binding cassette transporter
ABCB1: ATP-binding cassette subfamily B member 1
ABCG2: ATP-binding cassette subfamily G member 2
ACC: acetyl-CoA carboxylase
ACLY: ATP-citrate lyase
ADCC: antibody-dependent cell-mediated cytotoxicity
ALK: anaplastic lymphoma kinase
ALCL: anaplastic large cell lymphoma
AML: acute myeloid leukemia
BC: breast cancer
Bcl-2: B-cell lymphoma 2
BLCA: bladder cancer
BMDC: bone marrow-derived cell
CAF: cancer-associated fibroblast
CAIX: carbonic anhydrase IX
CAR: chimeric antigen receptor
CML: chronic myeloid leukemia
CRC: colorectal cancer
CTC: circulating tumor cell
EC: esophageal cancer
ECAR: extracellular acidification rate
ECM: extracellular matrix
EGFR: epidermal growth factor receptor
EMT: epithelial-mesenchymal transition
EV: extracellular vesicle
FAK: focal adhesion kinase
FASN: fatty acid synthase
FOXM1: forkhead box protein M1
FUT: fucosyltransferase
G6PD: glucose-6-phosphate dehydrogenase
GLUT: glucose transporter
HCC: hepatocellular carcinoma
HIF: hypoxia-inducible factor
HK2: hexokinase 2
HKDC1: hexokinase domain containing 1
HNSCC: head and neck squamous cell carcinoma
HSP: heat shock protein
Ig: immunoglobulin
LDH: lactate dehydrogenase
LUAD: lung adenocarcinoma
MAPK: mitogen-activated protein kinase
MCT: monocarboxylate transporter
MET: mesenchymal-epithelial transition
MMP: matrix metalloproteinase
mTORC1: mammalian target of rapamycin complex 1
NK cell: natural killer cell
NSCLC: non-small cell lung cancer
OAT: ornithine aminotransferase
OCR: oxygen consumption rate
OXPHOS: oxidative phosphorylation
PCa: prostate cancer
PDAC: pancreatic ductal adenocarcinoma
PD-L1: programmed death-ligand 1
PFK: phosphofructokinase
PHGDH: phosphoglycerate dehydrogenase
PI3K/AKT: phosphatidylinositol 3-kinase/protein kinase B
PKM2: pyruvate kinase M2
PMN: pre-metastatic niche
PTM: post-translational modification
PYCR1: pyrroline-5-carboxylate reductase 1
RAP1: Ras-related protein 1
RAS: reactive oxygen species
RCC: renal cell carcinoma
SDH: succinate dehydrogenase
TAN: tumor-associated phenotypes
TCA: tricarboxylic acid
TKI: tyrosine kinase inhibitor
TME: tumor microenvironment
Treg: regulatory T cell
VEGF: vascular endothelial growth factor
